# Gut–Brain Inflammatory Pathways in Attention-Deficit/Hyperactivity Disorder: The Role and Therapeutic Potential of Diet

**DOI:** 10.3390/metabo15050335

**Published:** 2025-05-19

**Authors:** Naomi Lewis, Jim Lagopoulos, Anthony Villani

**Affiliations:** 1 School of Health, University of the Sunshine Coast, 90 Sippy Downs Dr., Sippy Downs, QLD 4556, Australia; naomi.lewis@research.usc.edu.au; 2 Thompson Institute, University of the Sunshine Coast, 12 Innovation Pkwy., Birtinya, QLD 4575, Australia; 3 Thompson Brain and Mind Healthcare, Eccles Blvd., Birtinya, QLD 4575, Australia; jlagopoulos@tbmh.org.au

**Keywords:** attention-deficit/hyperactivity disorder, Western diet, Mediterranean diet, gut microbiome, dysbiosis, inflammation, anti-inflammatory, antioxidant, omega-3s

## Abstract

Attention-deficit/hyperactivity disorder (ADHD) is a common childhood-onset neurodevelopmental disorder that often persists into adulthood, leading to various adverse outcomes. Its underlying pathology is multifactorial, involving neurotransmitter imbalances, gut microbiota alterations, and oxidative and inflammatory dysregulation. Diet, a key environmental modifier of gut ecology, is consistently poorer in individuals with ADHD, with multiple nutrients implicated in its pathophysiology. This review examines the role of specific nutrients such as omega-3 fatty acids, key micronutrients, and potentially harmful dietary components, as well as broader dietary patterns, particularly the Western diet and Mediterranean diet (MedDiet), in relation to ADHD symptoms. It also evaluates both whole-diet and supplement-based clinical interventions, supporting the growing recognition of nutrition as a safe and relatively affordable modifiable factor in ADHD management. Additionally, the biological mechanisms linking diet to ADHD are reviewed, highlighting strong evidence for the involvement of gut dysbiosis and inflammatory processes. Despite the well-documented antioxidant, anti-inflammatory, and microbiome benefits of the MedDiet, direct research investigating its role in ADHD remains limited. Most whole-diet approaches to date have focused on elimination diets, leaving a significant gap in understanding the potential role of the MedDiet in ADHD management. Therefore, this review outlines preliminary evidence supporting the MedDiet and its key components as modulators of ADHD-related biological pathways, indicating its potential as a therapeutic approach. However, further research is required to rigorously evaluate its clinical efficacy. Finally, the limitations of observational and interventional nutritional research in ADHD are discussed, along with recommendations for future research directions.

## 1. Introduction

Attention-deficit/hyperactivity disorder (ADHD) is a common childhood-onset neurodevelopmental disorder which can remain impairing for many individuals into adulthood [1,2]. In addition to symptoms of inattention and/or hyperactivity and impulsivity, which comprise the core clinical diagnostic criteria [2], individuals can also experience problems with executive functioning [3], emotional regulation [4,5,6], sleep [7,8], and gastrointestinal function [9]. Moreover, individuals with ADHD often experience significant challenges including difficulties with peer relationships, reduced academic and occupational achievement, higher rates of accidental injuries, and lower self-esteem and overall life satisfaction [10,11,12]. Additionally, they have a greater risk of mood, developmental, and anxiety disorders [13] and have higher rates of suicide attempts and injuries related to self-harm [14]. ADHD often manifests concurrently with other conditions such as autism spectrum disorder (ASD), tics, learning disorders, oppositional defiance disorder, conduct disorder, and mood and anxiety disorders [15,16]. Consequently, the economic burden of ADHD is significant, with annual socioeconomic costs in high-income countries ranging from $US356 million to over US$20 billion [17].

Although the aetiology of ADHD is not fully understood, the condition is known to be influenced by multiple polygenetic and environmental factors which interact during early development, causing heterogenous neuropathology and symptom profiles [10,18]. Heritability rates are an approximated 70% to 90% [19,20,21,22], yet only a limited number of genes have been associated with ADHD, all of which only contribute a small risk [21,23]. Multiple environmental risk factors are also implicated in ADHD, including prenatal and perinatal factors such as maternal alcohol consumption and smoking, maternal genitourinary infection and pre-eclampsia, Caesarean-section delivery, formula feeding, low birth weight, prematurity, poorer maternal health, maternal stress and depression, extreme early adversity, and exposure to environmental toxins [18,24,25,26,27,28,29,30,31,32,33,34,35]. Maternal obesity prior to pregnancy also increases the risk of ADHD [36,37,38]. The strength of evidence varies across these risk factors, however [39,40], and causation has not been established [2,41].

Neurotransmitter alterations, particularly the catecholamines, dopamine and norepinephrine, are central to the neuropathology of ADHD [42]. Both norepinephrine and dopamine are involved with neuromodulation of brain areas and circuits implicated in ADHD [42,43]. Serotonin deficiency has also been implicated in the pathology underlying hyperactivity and impulsivity in ADHD [44,45,46]. ADHD pathology additionally involves gut dysbiosis, inflammation, and oxidative stress, with gut microbiota differences found in individuals with ADHD potentially contributing to impaired intestinal barrier integrity, systemic inflammation, and neuroinflammation [47]. Accordingly, many prenatal and perinatal risk factors for ADHD also have the potential to impact the development of the gut microbiome [48,49,50,51,52,53,54,55]. As one of the most influential environmental modifiers of the gut ecology [56], diet has consistently been found to be of poorer quality in those with ADHD compared to healthy controls [57]. This suggests not only that dysbiosis could be associated with mechanisms underpinning ADHD, but that nutritional factors may also be involved.

Although the negative effects of ADHD can persist throughout an individual’s lifespan, appropriate management can substantially improve core symptoms [58]. Medication remains the gold standard for ADHD treatment, demonstrating significantly higher short-term effectiveness in reducing symptoms compared to placebo [59,60,61]. First-line treatments for ADHD are stimulant medication such as methylphenidate and amphetamine, which work by blocking the reuptake and/or increasing the presynaptic efflux of catecholamines, increasing their availability [3,62]. However, a significant proportion of patients with ADHD (20–35%) do not respond effectively to stimulants [63,64], and medication alone often does not fully address the broader and long-term concerns of many ADHD patients [3,65]. Furthermore, medications are not well tolerated in some cases [60] and are associated with adverse effects including reduced appetite, sleep disturbance, headaches, tics, seizures, psychotic symptoms, and increased blood pressure and heart rate [10,59,66,67,68,69]. As such, hesitance and nonadherence to stimulants are common challenges [23], highlighting the need for safe and effective alternatives.

Nonpharmacological ADHD interventions are often employed as an adjunct therapy to pharmacological treatment, particularly when targeting specific functional impairments, and these represent important options for patients who refuse or are unable to take stimulants [3,65,67]. For example, cognitive behavioural therapy [70], mindfulness-based therapy [71], and aerobic exercise [72] have all been shown to reduce symptoms of inattention, impulsivity, and hyperactivity in those with ADHD.

Nutritional approaches may also offer effective, safe, and low-cost adjunct therapy for ADHD [73]. Meta-analyses have reported that a low-diet quality (e.g., high in ultra-processed foods) increases the risk of ADHD in both children and adults; in contrast, a diet consistent with high intake of fruits, vegetables, wholegrains, and fish may protect against ADHD [57,74]. Notably, the Mediterranean diet (MedDiet), which reflects the traditional eating habits of nations surrounding the Mediterranean Sea, is a well-known dietary pattern associated with optimal health and longevity [75]. Many of the observed health benefits are related to its antioxidant and anti-inflammatory qualities [76] and positive impacts on the gut microbiome [77]. However, despite rising evidence for the involvement of diet, inflammatory processes, and the gut microbiota in ADHD aetiology, there is limited research investigating the potential benefits of a MedDiet on risk for ADHD and severity of symptoms.

This narrative review examines the relationship between nutrient intake, dietary patterns—focusing on Western versus Mediterranean diets—and the diagnosis and symptoms of ADHD. This review also reports on outcomes of nutritional interventions in ADHD, highlighting biological pathways linking diet and ADHD pathophysiology. The potential benefits of the MedDiet will also be discussed, focusing on research that supports its positive effects on key biological pathways associated with ADHD. Lastly, gaps in current knowledge are identified, and important directions for future research outlined.

## 2. Key Nutrient and Dietary Patterns in ADHD and Related Interventions

### 2.1. Omega-3 Poly-Unsaturated Fatty Acids

Omega-3 poly-unsaturated fatty acids (PUFAs) have attracted considerable interest for their potential role in managing ADHD symptoms. Long-chain omega-3 PUFAs, namely eicosapentaenoic acid (EPA) and docosahexaenoic acid (DHA), are obtained only from the diet and are essential for a variety of processes, including maintenance of optimal brain structure and function and anti-inflammatory processes [78,79]. In recent decades there has been a shift in the proportions of PUFAs consumed in developed countries, including a marked reduction in omega-3s, which is posited as contributing to the pathogenesis of many diseases [80]. Children with ADHD have been shown to consume fewer omega-3-rich foods relative to healthy controls [81]. Further, two meta-analyses reported lower levels of blood/buccal cell omega-3 PUFAs in children and adolescents with ADHD compared to healthy controls and found that supplementation in ADHD modestly improved clinical symptoms from an investigation of sixteen [82] and eight [83] omega-3 randomised controlled trials (RCTs). One of these meta-analyses included some trials outside the scope of ADHD, however [82], warranting caution in interpreting findings specific to ADHD. A subsequent study found that lower blood levels of omega-3s and total PUFAs in children with ADHD were associated with higher ADHD symptoms and lower global functioning and quality of life [84].

Not all study reviews have found PUFA supplementation to effectively reduce ADHD symptoms. A 2019 systematic review of seven RCTs investigating the efficacy of omega-3 PUFAs demonstrated only a non-significant reduction in symptoms in children and adolescents with ADHD [85]. This review restricted outcome measures to the Conners’ ADHD rating scales, however, potentially limiting findings. Systematic reviews and meta-analyses of PUFA supplementation RCTs in children and adolescents with ADHD conducted in 2021 (including 31 studies) [86] and 2023 (including 36 studies) [87] found no significant benefit regarding core symptoms, behavioural difficulties, and quality of life [86], and only limited evidence for ADHD symptom improvement [87]. These studies included omega-3, omega-6, or a combination of both PUFAs in their analyses; however, overall outcomes were not differentiated by PUFA type. One review did not report the PUFA type or dosage used in each study [86]. However, a subgroup analysis in the other review compared fourteen omega-3 trials to two combined omega-3/omega-6 trials, finding no significant difference in parent-rated ADHD symptom outcomes [87], potentially reducing the perceived relevance of specific PUFA type. Nonetheless, the limited nature of this single comparison, which did not isolate the effects of omega-6 PUFAs, constrains the interpretation of individual effects of omega-3 PUFAs, and consequently the interpretation of primary outcomes derived from the combined PUFA analysis. See Table 1 for a summary of the key features and outcomes of these reviews. Notably, there is considerable variation in trial duration and supplement dosage within reviews.

Overall, findings from systematic investigations of omega-3 PUFA supplementation have been inconsistent, complicated by heterogeneity in methodology. However, relatively recent omega-3 RCTs have demonstrated effectiveness in reducing various ADHD symptoms [88,89], including two trials not included in the discussed meta-analyses [90,91] and one reporting outcomes comparable with those achieved with methylphenidate [92]. Notably, benefits may be most significant in those where baseline EPA levels are low [93]. In one of the few dietary studies of adults with ADHD, a randomised crossover omega-3 PUFA supplementation trial was also positive, demonstrating improved ADHD symptoms in individuals with comorbid ADHD and ASD and improved attention and working memory in those with ASD without ADHD [91]. Conversely, other studies have reported minimal or no improvement in ADHD symptoms following omega-3 supplementation across most outcome measures [94,95,96], with potential for a negative effect when baseline levels of EPA are high [93].

The variability in findings across studies may be attributed to methodological and reporting inconsistencies [86,87], as well as differences in study design, duration, outcome measures, supplement composition, and, in one meta-analysis, clinical group [82]. As shown from summaries of the discussed omega-3 studies presented in Table 1, individual trials involving child and adolescent participants with predominantly positive outcomes [88,89,90,92,93] generally employed higher daily dosages of omega-3 PUFAs (775 mg to 1300 mg) compared to trials reporting neutral or negative outcomes [94,95,96] (420 mg to 840 mg). Trial durations however, do not appear to differ by outcome. Additionally, baseline omega-3 deficiency likely influences response to supplementation, with lower levels potentially leading to more pronounced benefits, as noted in one study [93]. Similarly important but not sufficiently investigated, an imbalance of omega-3 and omega-6 PUFAs may also be a significant feature of ADHD influencing supplementation outcomes. EPA, for example, possesses anti-inflammatory properties that are vital in countering the pro-inflammatory properties of the omega-6 PUFA arachidonic acid (ARA) [97]. Elevated omega-6:omega-3 ratios have been reported in ADHD compared to healthy controls [97,98] and have been found to be positively associated with ADHD symptoms and inversely associated with quality of life in children with ADHD [84]. As such, conflicting findings may also reflect variability in balancing of the omega-6:omega-3 ratio through supplementation, which may be as critical to symptom improvement as correcting an omega-3 deficiency itself.

While further research is needed, lower omega-3 PUFA intake in children with ADHD [81], which may explain observed deficiencies [82,83], suggests supplementation could improve symptoms in deficient individuals [93]. However, when evaluating the use of omega-3 PUFAs in ADHD, whether in research or clinical settings, it is essential to assess dietary intake and/or blood levels to identify any existing deficiencies or imbalances, including the proportions of various PUFAs, and determine the optimal dosage, for maximum benefits.

### 2.2. Micronutrients

A growing body of evidence also highlights deficiencies across various micronutrients in ADHD, which may be associated with poor overall diet quality (see Section 2.3), whilst the efficacy of vitamin and mineral supplementation in ADHD has been assessed via single or multiple nutrient approaches. Low intake of and deficiencies in the essential minerals zinc, magnesium, and iron in particular are consistently reported in those with ADHD, and have therefore become a focus of targeted supplement trials. A case-control study reported reduced levels of zinc, magnesium, and copper in both serum and hair samples from children with ADHD, with low levels of zinc and magnesium associated with symptoms of inattention, hyperactivity, and impulsivity [99]. Low serum ferritin levels have been observed in children with ADHD compared to healthy controls, with levels inversely related to ADHD severity [100]. Ferritin levels were twice as low in the ADHD group, whilst a third of ADHD participants had extremely low levels indicating low iron stores [100]. Studies have also shown that children with ADHD have lower brain iron content compared to healthy controls [101]. A 2019 meta-analysis of seven magnesium studies found that individuals with ADHD had significantly lower serum magnesium levels than healthy controls [102]. Similarly, a 2024 review of observational, interventional, and meta-analytic research investigating magnesium in ADHD in the previous 5 years confirmed that lower hair and serum magnesium levels frequently occur in children with ADHD [103].

Clinical trials have provided additional insight into the role of these minerals in ADHD. A meta-analysis and qualitative synthesis of research on iron supplementation in participants aged under 25 years with ADHD found that supplementation may improve ADHD symptoms in those with iron deficiency; however, the overall findings were limited by underpowered studies and low-quality evidence [104]. A single-arm iron supplementation trial published subsequently to this meta-analysis found a significant improvement in inattention and hyperactivity symptoms after 6 weeks of supplementation in children with ADHD (*N* = 32), including those who were not deficient in iron at baseline (*n* = 23) [105]. A meta-analysis of six RCTs of zinc supplementation in children with ADHD found a reduction in overall ADHD scores [106]. Hyperactivity and inattention scores were not reduced following supplementation; however, the analysis did not account for baseline zinc levels or for existing zinc deficiencies [106]. Despite magnesium’s relevance in ADHD, studies examining its effectiveness as a standalone treatment are limited. Though the 2024 review claimed magnesium supplementation has potential for improving ADHD symptoms, it concluded that a causal link has yet to be established [103]. In a study of 50 children with ADHD and magnesium deficiency, those receiving 6 months of magnesium supplementation showed a significant decrease in hyperactivity symptoms and an increase in hair magnesium levels compared to baseline and the control group [107]. Similarly, an 8-week trial of magnesium supplementation in children and adolescents with both ADHD and magnesium deficiency found significant improvements in hyperactivity, impulsivity, inattention, oppositional behaviour, and conceptual reasoning in those receiving the magnesium (*n* = 9) compared to standard medical treatment (*n* = 9) [108].

Among vitamins, vitamin D and B group vitamins are commonly implicated in ADHD. Folic acid and vitamin B12 were found to be lower in ADHD [98,109,110,111], and an inverse relationship between vitamin B12 levels and ADHD symptoms has been observed in one study [110] but not another [111]. A 2018 systematic review and meta-analysis of 13 studies found that children and adolescents with ADHD had lower serum 25-hydroxyvitamin D3 levels compared to healthy controls, with lower levels significantly associated with an increased likelihood of ADHD and suboptimal perinatal levels linked to later childhood ADHD diagnosis [112]. A recent study also reported significantly lower levels of serum 25-hydroxyvitamin D3 in children with ADHD, along with a low dietary intake of vitamin D compared to children without ADHD [113]. This finding suggests a diet-related vitamin D deficiency in ADHD.

Research on vitamin monotherapy in ADHD has largely focused on vitamin D. A 2019 systematic review and meta-analysis of four RCTs investigating vitamin D supplementation as an adjunct to methylphenidate in ADHD showed increased serum vitamin D levels alongside a modest but significant improvement in total ADHD symptom scores, inattention, hyperactivity, and overall behaviour [114]. However, no significant changes were observed in oppositional behaviour scores [114]. A recent 8-week vitamin D plus neurofeedback RCT in 35 children with ADHD similarly demonstrated increased serum levels with vitamin D supplementation compared to placebo, but that supplementation did not improve ADHD symptoms, yet together with neurofeedback improved brain wave patterns [115]. An 8-week RCT combining vitamin D and magnesium in 66 children with ADHD and low baseline serum levels demonstrated an increase in serum levels of vitamin D and magnesium and reduced emotional, peer, externalising, and total difficulties scores, but not hyperactivity, conduct, prosocial, or externalising scores on one scale [116] and reduced conduct, social, and anxiety/shy scores, but not psychosomatic problems, on another scale [117], compared to controls.

Finally, ADHD supplement research has also examined broad-spectrum micronutrient formulations, typically comprising combinations of vitamins, minerals, amino acids, and antioxidants. A 10-week RCT of a broad-spectrum micronutrient in children with ADHD improved overall functioning, inattention, emotional regulation, and aggression, but not hyperactive/impulsive symptoms, in those assigned to the micronutrient group (*n* = 47) compared to placebo (*n* = 46) [118]. An 8-week RCT of a broad-spectrum micronutrient formula in adults with ADHD resulted in significant improvements in self- and observer- but not clinician-rated ADHD symptoms for the micronutrient group (*n* = 42) compared to the placebo group (*n* = 38) [119]. Furthermore, while the improvements observed with the broad-spectrum micronutrient supplementation were significant during the trial, many outcomes regressed towards baseline levels over a 1-year follow-up period, though not completely returning to initial levels. Notably, participants who continued with the supplementation maintained their improvements [120]. Both broad-spectrum micronutrient studies reported significant group differences in serum vitamin D, B12, and folate levels at the intervention end point, which increased from baseline in the micronutrient groups [118,119]. An open-label reversal trial in 14 children with ADHD undergoing two 8-week micronutrient treatment phases and two 4-week treatment withdrawal phases found improved ADHD symptoms, mood, and overall functioning during treatment phases and deterioration in withdrawal phases [121]. In contrast, an 8-week micronutrient RCT in children with ADHD and irritability showed clinician-rated global improvement; however, there was no significant improvement in core ADHD symptoms in those receiving micronutrients (*n* = 71) compared to placebo (*n* = 55) [122]. This suggests that, while micronutrients may contribute to overall wellbeing or emotional regulation, their direct impact on ADHD symptomatology remains uncertain.

**Table 1:** Summary of supplement interventions in ADHD.

Study	Study Type	Sample Size/Population	Supplement and Dosage ^†^	ADHD Medication	Duration	Primary Outcomes	Key Findings
Hawkey and Nigg (2014) [82]	Meta-analysis of RCTs	16 studies (*N* = 1408, age range 6–18 years, *M* = 9.7 years, with or without ADHD)	Omega-3 (total: 120–2513 mg; EPA: 0–1373 mg; DHA: 0–1140 mg)	In two studies, supplementation was an adjunct to ADHD medication	7–24 weeks (*M* = 14.5 weeks)	ADHD symptom severity (parent- and teacher-rated; pooled ratings)	Significant reduction in hyperactivity-impulsivity (parent, teacher, pooled ratings) and inattention (parent and pooled ratings), compared to placebo.
Chang et al. (2018) [83]	Meta-analyses of RCTs	First meta-analysis (1): seven studies (*N* = 534). Second meta-analysis (2): three studies (*N* = 214). Age range 4–17 years	1: Omega-3 (total: 120–1290 mg; EPA: 80–650 mg; DHA: 0–640 mg) 2: Omega-3 (total: 122–345 mg; EPA: 0–153 mg; DHA: 29–345 mg)	Not reported	Not stated	1: ADHD clinical symptom scores (parent- and teacher-rated) 2: Cognitive measures associated with attention	1: Significant reduction in parent-rated total, inattention, and hyperactivity symptoms; no effect on teacher-rated symptoms compared to placebo. 2: Significant improvement in omission and commission errors; no improvement in memory or information processing compared to placebo.
Abdullah et al. (2019) [85]	Systematic review of RCTs	Six studies (*N* = 564, age range 3–18 years)	Omega-3 (total: 100–1400 mg; EPA: 100–1000 mg; DHA: 0–400 mg)	In two studies, supplementation was an adjunct to ADHD medication	8 weeks–4 months	ADHD symptom severity (parent- and/or teacher-rated; CPRS, CTRS)	Small, non-significant reduction in ADHD symptoms compared to placebo in five of six studies.
Händel et al. (2021) [86]	Meta-analysis of RCTs	24 studies (*N* = 1755, age range 6–18 years)	Omega-3 and/or Omega-6 (PUFA type and dosage not reported)	In three studies, supplementation was an adjunct to ADHD medication	8 weeks–12 months	ADHD symptom severity (parent- and teacher-rated)	No significant difference in ADHD symptoms based on parent or teacher ratings compared to placebo/control.
Gillies et al. (2023) [87]	Systematic review of RCTs and quasi-randomised trials	36 studies (*N* > 2374) children and adolescents under 18 years	Omega-3 (EPA, DHA, ALA; 19 trials), Omega-6 (AA, LA, GLA; 2 trials), Combined Omega-3/6 (6 trials), Omega-3 + co-interventions (8 trials), Omega-3/6 + medication (1 trial) (EPA [22 studies]: 33–1039 mg; DHA [22 studies]: 2.7–3600 mg); ALA [2 studies]: 60–1080 mg; AA [2 studies]: 40–60 mg; LA [5 studies]: 240–360 mg; GLA [8 studies]: 6–345 mg)	In five omega-3 and one omega-3/6 trials, supplementation was an adjunct to ADHD medication	2 weeks–6 months	ADHD symptom severity (parent-, teacher-, and clinician-rated)	Low-certainty evidence for improvement in ADHD symptoms. High-certainty evidence for no effect on parent-rated total, inattention, and hyperactivity/impulsivity symptoms.
Bos et al. (2015) [88]	Double-blind RCT	40 boys with ADHD aged 8–14 years (*M* = 10.3 years): 20 intervention/20 placebo; 39 matched typically developing controls (*M*_age_ = 10.9 years): 20 intervention/19 placebo	Omega-3 (total: 1300 mg; EPA: 650 mg; DHA: 650 mg)	38 of 40 ADHD participants were taking ADHD medication	16 weeks	ADHD symptom severity (parent-rated; CBCL, SWAN)	Reduced inattention symptoms on the CBCL in both ADHD and control groups compared to placebo; no effect observed on the SWAN scale.
Rodríguez et al. (2019) [89]	Double-blind RCT	66 participants aged 6–18 years (*M* = 11.7 years): 32 intervention/34 placebo	Omega-3 (total: 1240 mg; EPA: 90 mg; DHA: 1000 mg; DPA: 150 mg)	24 of 32 participants in the intervention group and 24 of 34 in the placebo group were taking ADHD medication	6 months	ADHD symptom severity (parent-rated; EDAH, CPRS)	Reduced inattention, hyperactivity, and overall ADHD scores on the EDAH scale compared to placebo. No between-group differences on CPRS scores.
San Mauro Martin et al. (2022) [90] *	Controlled trial	31 participants aged 6–16 years (*M* = 10.7 years): 13 intervention/18 placebo	Omega-3 (total: 775 mg; EPA: 550 mg; DHA: 225 mg)	Not reported	8 weeks	Impulsivity (child-report; BIS-11c)	Significantly lower levels of impulsivity compared to controls.
Dashti et al. (2014) [92]	Double-blind RCT	85 participants aged 6–12 years (*M* = 8.2 years): 29 Ritalin/28 omega-3/28 placebo	Omega-3 (total: 1000 mg). A second intervention group received Ritalin	ADHD-medication-naïve	Duration not clearly reported	ADHD symptom severity (parent- and teacher-rated; CPRS, CTRS)	Significant reduction in ADHD symptom severity in both the omega-3 and Ritalin groups compared to placebo. No significant difference in symptom reduction between treatment groups.
Chang et al. (2019) [93]	Double-blind RCT	92 participants aged 6–18 years: 48 intervention/44 placebo	Omega-3 (EPA only: 1200 mg)	ADHD-medication-naïve or medication-free for the past 6 months	12 weeks	Blood PUFA levels; ADHD symptoms (CPT)	Greater improvement in focused attention (variability) in EPA group compared to placebo. Greater improvement in vigilance and focused attention (hit reaction time) in EPA group with lowest baseline EPA compared to placebo. Less improvement in impulsivity (commission errors) in EPA group than placebo. Less improvement in ADHD and emotional symptoms in EPA group with highest baseline EPA compared to placebo.
Lundbergh et al. (2022) [91] *	Double-blind randomised cross-over trial	26 participants aged 18–40 years (*M* = 28 years) with ASD (14 with comorbid ADHD)	Omega-3 (total: 4000 mg; EPA: 2400 mg; DHA: 1600 mg)	Some participants were taking ADHD medication	Two 1-month supplement periods (omega-3 and placebo)	Sustained attention (d2-test); spatial working memory (Corsi); ADHD symptoms (adult-rated; CAARS)	Improvement in working memory and sustained attention compared to placebo. Participants with comorbid ADHD showed improvements in ADHD symptom scores.
Cornu et al. (2018) [94]	Double-blind RCT	162 participants aged 6–15 years (*M* = 9.9 years): 80 intervention/82 placebo	Omega-3 (ages 6–8: total: 420 mg; EPA: 336 mg; DHA: 84 mg; ages 9–11: total: 630 mg; EPA: 504 mg; DHA: 126 mg; ages 12–15: total: 840 mg; EPA: 672 mg; DHA: 168 mg)	No ADHD medication for 1 month before and during the trial	3 months	ADHD symptom severity (parent-rated; ADHD-RS-IV)	Greater reduction in ADHD symptoms observed in the placebo group compared to the intervention group.
Crippa et al. (2019) [95]	Double-blind RCT	50 participants aged 7–14 years (*M* = 11.0 years): 25 intervention/25 placebo	Omega-3 (DHA only: 500 mg)	ADHD-medication-naïve	6 months	ADHD symptom severity (parent-rated; ADHD-RS-IV)	Improvement in hyperactivity/impulsivity and total ADHD symptoms observed in both groups, with no significant difference between groups.
Widenhorn-Müller et al. (2014) [96]	Double-blind RCT	95 participants aged 6–12 years (*M* = 8.9 years): 46 intervention/49 placebo	Omega-3 (total: 720 mg; EPA: 600 mg; DHA: 120 mg)	No ADHD medication for 6 months before and during the trial	16 weeks	ADHD symptom severity (teacher- and parent-rated; DISYPS-II)	No significant changes in teacher- or parent-rated ADHD symptom scores compared to placebo.
Elliott et al. (2024) [104]	Meta-analysis of RCTs	Three studies (*N* = 124) for hyperactivity and two trials (*N* = 75) for inattention (participants under 25 years with low serum iron or iron deficiency)	Iron (ferrous sulphate: 80 mg or 300 mg; or ferrous fumarate: 200 mg if <30 kg; 400 mg if >30 kg)	In one study, supplementation was an adjunct to ADHD medication	12 weeks	ADHD symptom severity (parent- and teacher-rated)	Non-significant improvements observed in hyperactivity and inattention scores compared to placebo.
Kumar et al. (2024) [105] *	Single-arm intervention	32 children aged 4–12 years (*M* = 8.2 years; 23 were ferritin sufficient)	Iron (3 mg/kg)	ADHD-medication-naïve	6 weeks	ADHD symptom severity (parent- and teacher-rated; CPRS, CTRS). Attention performance (CCTT)	Significant improvement in parent-rated hyperactivity and inattention; no significant change in teacher-rated symptoms. Significant improvement in inattention on CCTT.
Talebi et al. (2022) [106]	Meta-analysis of RCTs	Six studies (*N* = 489, age range 7–10 years)	Zinc (10–40 mg)	In four studies, supplementation was an adjunct to ADHD medication	6–12 weeks	ADHD symptom severity (parent- and teacher-rated)	Significant reduction in total ADHD scores compared to control, but no significant changes on subscales.
Starobrat-Hermelin et al. (1997) [107]	RCT	75 participants aged 7–12 years with magnesium deficiency: 50 intervention/25 control	Magnesium (approximately 200 mg)	Not reported	6 months	ADHD symptom severity (parent- and teacher-rated; CPRS, CTRS)	Increased hair magnesium levels and decreased hyperactivity symptoms compared to control.
El Baza et al. (2016) [108]	RCT	18 participants aged 6–16 years (*M* = 7.7 years) with magnesium deficiency: 9 intervention/9 control	Magnesium (200 mg)	All participants were taking ADHD medication	8 weeks	ADHD symptom severity (parent-rated; CPRS)	Improvement in hyperactivity, impulsivity, and inattention compared to control.
Gan et al. (2019) [114]	Meta-analysis of RCTs	Four studies (*N* = 256, age range 2–18 years)	Vitamin D (1000 IU/day–50,000 IU/week)	In all studies, supplementation was an adjunct to ADHD medication	6 weeks–3 months	ADHD symptom severity (clinician-, teacher-, and parent-rated)	Small improvements in ADHD total scores, inattention, hyperactivity, and behaviour scores compared to control. No improvement in oppositional scores.
Mirhosseini et al. (2024) [115] *	Double-blind RCT	35 participants aged 7–13 years (*M* = 9.2 years) receiving neurofeedback therapy: 20 intervention/15 placebo	Vitamin D (50,000 IU/week)	No ADHD medication for one year before and during the trial	2 months	Brain wave patterns (EEG); ADHD symptom severity (CPRS, ADHD-RS-IV)	Improvement in electrophysiological (EEG) measures; no improvement in ADHD symptom scores compared to placebo.
Hemamy et al. (2021) [116]	Double-blind RCT	66 participants aged 6–12 years (*M* = 9.1 years) with vitamin D and magnesium deficiency: 33 intervention/33 placebo	Vitamin D (50,000 IU/week) and magnesium (6 mg/kg)	No difference in ADHD medication dose between groups	8 weeks	Mental health status (parent-rated; SDQ)	Reduction in emotional, peer, internalizing problems, and total difficulties compared to placebo. No differences in conduct, hyperactivity, or prosocial behaviour.
Hemamy et al. (2020) [117]	Same study as above					Behaviour problems (parent-rated; CPRS-48)	Reduction in conduct, social, and anxiety/shyness scores, but not psychosomatic problems, compared to placebo.
Rucklidge et al. (2018) [118]	Double-blind RCT	93 participants aged 7–12 years (*M* = 9.8 years): 47 intervention/46 placebo	Broad-spectrum micronutrient formula (“Daily Essential Nutrients”: 13 vitamins, 17 minerals, four amino acids; titrated to 12 or 15 capsules)	No ADHD medication for 4 weeks before and during the trial	10 weeks	ADHD symptom severity (clinician-rated ADHD-RS-IV; parent-rated CPRS-R:L); Clinical Global Impression-Improvement (CGI-I)	Improvement in overall functioning and inattention (CGI-I-ADHD scale), reduced impairment, and improved emotional regulation and aggression compared to placebo. In total, 32% on micronutrients showed clinical improvement in inattentive symptoms compared to 9% on placebo (CGI-I). No significant differences in clinician-, parent-, or teacher-rated ADHD symptoms from other measures between groups.
Rucklidge et al. (2014) [119]	Double-blind RCT	80 participants aged 16 years or older (*M* = 35.2 years): 42 intervention/38 placebo	Broad-spectrum micronutrient formula (EMPowerPlus: 36 ingredients; titrated to 15 capsules)	No ADHD medication for 4 weeks before and during the trial	8 weeks	ADHD symptom severity (self-, clinician-, and observer-rated; CAARS); Clinical Global Impression-Improvement (CGI-I)	Improvement in ADHD symptoms based on self- and observer-report, but not clinician-report. Improved clinician-rated global functioning and ADHD symptoms (from CGI-I) compared to placebo.
Gordon et al. (2015) [121]	Open-label reversal design intervention	14 participants aged 8–12 years (*M* = 10.2 years)	Broad-spectrum micronutrient formula (EMPowerPlus: 36 ingredients; titrated to 15 capsules)	No ADHD medication for 4 weeks before and during the trial	Two 8-week treatment phases with 4-week washout periods	ADHD symptom severity (parent-rated CPRS-R:L); mental health status (parent-rated; SDQ); Clinical Global Impression-Improvement (CGI-I)	Improvement in ADHD symptoms, mood, and overall functioning during intervention phases; deterioration during washout periods. At the end of the second treatment phase, 79% rated as “much improved” or “very much improved” on overall clinical impression ratings; lower total difficulties, conduct problems, hyperactivity, and impact score and improved prosocial behaviour.
Johnstone et al. (2022) [122]	Double-blind RCT	126 participants with irritability aged 6–12 years (*M* = 9.8 years): 71 intervention/55 placebo	Broad-spectrum micronutrient formula (“Daily Essential Nutrients”: 13 vitamins, 17 minerals, four amino acids; titrated to 9 or 12 capsules)	No ADHD medication for 4 weeks before and during the trial	8 weeks	Composite of ADHD symptoms, oppositional defiant disorder, disruptive mood dysregulation, peer conflict, and impairment (parent-rated CASI-5); Clinical Global Impression-Improvement (CGI-I)	Both groups showed reduced CASI-5 scores; no significant difference between groups. 54% of the micronutrient group and 18% of the placebo group were responders based on CGI-I scores.

AA = arachidonic acid; ADHD = attention-deficit/hyperactivity disorder; ADHD-RS-IV = Attention-Deficit/Hyperactivity Disorder Rating Scale-Fourth Edition; ALA = alpha-linolenic acid; ASD = autism spectrum disorder; BIS-11c = Barratt Impulsiveness Scale; CAARS = Conners’ Adult ADHD Rating Scale; CASI-5 = Child and Adolescent Symptom Inventory, Fifth Edition; CBCL = Child Behavior Checklist; CCTT = Children’s Color Trails Test; CGI-I = Clinical Global Impression–Improvement; CPRS = Conners’ Parent Rating Scale; CPRS-48 = Conners’ Parent Rating Scale–48 items; CPRS-R:L = Conners’ Parent Rating Scale–Revised: Long Version; CPT = Continuous Performance Test; CTRS = Conners’ Teacher Rating Scale; DHA = docosahexaenoic acid; DISYPS-II = Diagnostic System for Mental Disorders in Children and Adolescents, Second Edition; DPA = docosapentaenoic acid; EDAH = Evaluation of Attention Deficit with Hyperactivity; EEG = electroencephalography; EPA = eicosapentaenoic acid; GLA = gamma-linolenic acid; IU = international units; LA = linoleic acid; PUFA = polyunsaturated fatty acid; RCT = randomised controlled trial; SDQ = Strengths and Difficulties Questionnaire; SWAN = Strengths and Weaknesses of ADHD Symptoms and Normal Behavior Scale. All participants had ADHD unless specified otherwise. * Individual trial not included in any of the reviewed meta-analyses or systematic reviews for the corresponding nutrient (i.e., omega-3, iron, vitamin D). ^†^ All dosages are per day, apart from where indicated as per week (vitamin D).

There is emerging evidence supporting nutrient supplementation for individuals with ADHD, particularly, but not always, for those with a prior deficiency. Further research is needed to clarify changes in serum nutrient levels from pre- to post-intervention, comparing ADHD patients who are deficient to those who are not. Regarding broad-spectrum micronutrients, since only some nutrients have shown increased serum levels following treatment, and given that the cost associated with the supplement contributed to discontinuation [120,123], identifying those nutrients which are associated with greatest symptomatic improvement could improve long-term feasibility of supplementation. Table 1 provides a summary of the supplement intervention studies included in this review.

### 2.3. Detrimental Dietary Patterns and Components and Elimination Diets

In contrast to the diets of our ancestors, which were rich in minimally processed animal- and plant-based foods [124], the modern Western diet consists primarily of ultra-processed items, including processed meats, sugar-sweetened beverages (SSBs), commercial sweet and savoury snacks, processed grains and fast food, and red meat [125]. This dietary pattern is characterised by high total and saturated fat, refined sugars, and sodium, and low omega-3 PUFAs, dietary fibre, micronutrients, and antioxidants [125,126].

The nutrient-poor profile of modern Western diets is associated with chronic inflammation, impaired gut health, and weakened immunity [127], with serious related health consequences including obesity, Type 2 diabetes, cardiovascular disease (CVD), and certain cancers [124]. The Western dietary pattern is also associated with higher risk of developing psychological symptoms and mental ill-health [128,129,130,131]. Higher consumption of saturated fat and sugar has been associated with impaired memory and executive functioning [132], whilst adherence to a Western-style dietary pattern has been associated with decreased left hippocampal volume, a brain region critical for learning and memory [133], and significant declines in hippocampal-dependent learning and memory in healthy individuals [134].

Poor-quality diets and their components are also linked to ADHD. Children in the top quintile for a Western dietary pattern were more likely to have ADHD compared to those in the lowest quintile, while a healthy dietary pattern was inversely associated with ADHD [135]. In similar studies of children [136] and adolescents [126], a Western dietary pattern was positively associated with ADHD diagnosis; however, there was no association between ADHD diagnosis and a “healthy” dietary pattern among adolescents [126]. ADHD diagnosis has also been associated with higher intake of saturated fat [137], lower adherence to a MedDiet (discussed in Section 2.4), and higher intake of ultra-processed foods and SSBs in children [138] and in both children and adolescents [139,140,141]. Children and adolescents in the highest quartile of adherence to a combined Mediterranean and Dietary Approaches to Stop Hypertension (DASH) diet were found to have significantly lower odds of developing ADHD compared to those in the lowest quartile [142]. Additionally, a recent systematic review and meta-analysis showed that, whilst poor diet quality is linked with an increased risk of ADHD in both children and adults, a dietary pattern rich in fruits, vegetables, and fish may protect against ADHD [57].

The severity of ADHD symptoms also varies in relation to dietary patterns. ADHD symptom scores of children following a dietary pattern high in ultra-processed foods were greater than those of children following a diet high in fruits, vegetables, and dairy [143]. A prospective study found that a “sweet” dietary pattern, high in ultra-processed snacks, chocolate, sweet drinks, and ice cream, at 4 years of age was associated with a higher risk of ADHD symptoms at 6 years, whereas the opposite trend was observed for a dietary pattern rich in vegetables [144]. Another study similarly found that fruit and vegetable intake was inversely associated with inattention in children with symptoms of ADHD [145]. Greater learning and behavioural problems in addition to ADHD symptoms have also been associated with higher intakes of ultra-processed foods, whilst the reverse pattern was observed for a dietary pattern rich in vegetables and dairy [146]. Furthermore, adherence to a MedDiet was negatively correlated to impulsivity [147] and inattention [141] in children diagnosed with ADHD. Among a sample of adolescents with no specified conditions, however, only the diet quality index and the ideal diet score, but not the MedDiet score, were associated with attention [148].

Many studies have specifically explored the link between sugar consumption and ADHD risk. A dose-response relationship between SSB consumption and ADHD has been observed, with children who consumed seven or more servings of SSBs per week having a four-fold greater risk of ADHD than those consuming less than this amount [149]. A 2020 systematic review and meta-analysis reported a positive relationship between overall sugar and SSB consumption and symptoms of ADHD in children; however, dietary sugar consumption alone was not found to increase the risk of developing ADHD symptoms [150]. Another more recent study found that children and adolescents with ADHD consumed more sugar than their peers and observed positive correlations between ADHD symptoms and excessive consumption of ultra-processed sugary foods [137]. Conversely, one study found no association between sucrose consumption in children between 6 and 11 years and the incidence of ADHD [151].

Food additives and natural food chemicals are also thought to play a role in ADHD. Artificial food colourings (AFCs) in particular are often implicated in ADHD due to potential sensitivities in affected individuals. A 2004 meta-analysis of 15 clinical trials found that these food additives may promote hyperactivity in hyperactive children [152]. A 2020 systematic review investigating elimination diets in children with ADHD found benefits for attenuating ADHD symptoms in two of six AFC elimination interventions and ten of twelve elimination diet studies [153]. Elimination diets are designed to exclude specific food chemicals or items believed to exacerbate symptoms due to sensitivities, including additives such as AFCs and artificial flavours, as well as some natural chemicals such as salicylates [153]. A recent two-armed RCT comparing an elimination diet to an active control group involving 165 children (aged 5 to 12 years) who were randomised to either an elimination diet or healthy diet showed that, after 5 weeks, 51% of those in the healthy diet group experienced partial or full treatment response (reduced ADHD symptoms and emotion regulation problems) compared with only 35% of those in the elimination diet group [154]. Adherence to both diet interventions was similarly good-to-excellent, indicating that the observed benefits were unlikely due to the exclusion of food sensitivities. Instead, the findings suggest that following a healthy diet could serve as a promising complementary treatment in families with motivated parents [154].

Various open-label, single-arm elimination diet interventions have also recently been conducted in children with ADHD. A 5-week diet in 47 children with ADHD (aged 6 to 9 years) consisting of a limited number of hypoallergenic foods (including rice, potatoes, chicken, and meat, a range of vegetables, legumes, and fruits, and no added sugars, honey, gluten, egg, dairy, artificial food colours, preservatives, or additives) not only led to fewer participants being classified as obese or overweight, but also reduced impulsivity, hyperactivity, and learning problem scores [155]. In a 32- to 33-day elimination diet involving a limited selection of foods, 63% of 79 boys aged 8 to 10 years with ADHD showed a reduction by at least 40% in symptom scores which correlated with brain function measured through functional magnetic resonance imaging [156]. Similarly, after a 4-week elimination diet in children aged 7 to 13 years with ADHD (*N* = 28), there was a significant reduction in ADHD symptoms, with 17 participants having a reduction in symptoms by at least 40% and 10 considered non-responders, whilst 27 food sensitivity reactions were identified in a reintroduction phase [157]. Further, symptoms remained lower after approximately 3.5 years with subsequent individual nutritional recommendations, suggesting that an individually tailored diet may prove beneficial in ADHD in the long term [158]. Research has also implicated naturally occurring lectins in ADHD [159] and improvement of gastrointestinal and ADHD symptoms through the elimination of gluten among those with non-celiac gluten sensitivity [160,161].

### 2.4. The Mediterranean Diet and Related Diets

The MedDiet is typically defined as a plant-based dietary pattern, consistent with a high intake of fruits, vegetables, wholegrains, legumes, nuts, and seeds and liberal use of extra-virgin olive oil (EVOO) as the primary fat source; a moderate intake of fermented dairy, fish, and seafood; and an infrequent intake of red and processed meats, butter, and ultra-processed foods [162,163]. Being predominately plant-based, the MedDiet is naturally low in saturated fat, sugar, and sodium yet rich in functional nutrients including vitamins and minerals, carotenoids, unsaturated fatty acids, and phenolic compounds [164].

Health benefits attributed to the MedDiet are manifold and span both physical and psychological domains. A recent meta-analysis of over 12.8 million participants showed that higher adherence to a MedDiet was associated with a significant reduction in risk for CVD, coronary heart disease, myocardial infarction, overall cancer incidence, neurodegenerative diseases, diabetes, and overall mortality [75]. Moreover, adherence to a MedDiet has also been associated with a reduced risk of depression and cognitive impairment [165], slower rates of cognitive decline and decreased risk for Alzheimer’s disease in adults [166], higher self-perceived mental and physical health [167], higher health-related quality of life [168], as well as concentration and selective attention [169], in children, and better learning and memory performance in older adults [170]. MedDiet clinical trials have demonstrated a reduction in depression and improved health-related quality of life compared to a social intervention [171] and a reduction in depression in individuals with Type 2 diabetes [172]. 

While the observational studies outlined in the previous section have shown an inverse relationship between adherence to healthy dietary patterns (e.g., MedDiet/DASH diet) and the risk of developing ADHD, strong empirical evidence supporting the use of the MedDiet for management of ADHD symptoms is scant. An 8-week intervention in children and adolescents with ADHD aged 6 to 16 years did not significantly reduce impulsivity scores in those receiving a personalised MedDiet intervention (*n* = 12) compared to healthy controls following their usual diet (*n* = 21) [90]. However, the small sample size of the intervention group suggests that the study may not have been adequately powered to detect significant findings. Moreover, despite almost all participants having inattentive or combined ADHD, the investigators did not examine attention or other ADHD-related outcomes. A 12-week DASH diet has been investigated in children with ADHD aged 6 to 12 years [173]. Similar to the MedDiet, the DASH diet is a predominantly plant-based dietary pattern emphasising high quantities of fruits, vegetables, and low-fat dairy, with a limited intake of red and processed meats [173]. Children randomly assigned to the DASH diet (*n* = 40) had greater reductions in ADHD symptoms, emotional symptoms, conduct problems, peer relationship problems and improved prosocial behaviour compared to those assigned to the control diet (*n* = 40) [173].

To date, most clinical dietary trials in ADHD have involved elimination diets, and there is clear need for further robust research investigating the impact of healthy dietary patterns, including the MedDiet. While the current evidence is limited, the findings are mostly positive, with one study demonstrating superiority of a healthy diet over an elimination diet [154]. However, elimination diet interventions also reveal that food sensitivities may play a role in symptom severity for some with ADHD. Collectively, these findings highlight that diet should remain a key component of comprehensive ADHD management and patient care.

## 3. Key Diet-Regulated Biological Pathways

### 3.1. Dietary Influence on Gut Dysbiosis and Inflammation in ADHD 

Although there is a confluence of evidence linking ADHD to diet and nutrition, identifying the exact factors that underpin these relationships remains complex and challenging. The increasing prevalence of conditions such as ADHD has paralleled the increase in diet-related health issues, with exposure to nutritional factors during or even prior to pregnancy being important [174]. For example, pre-pregnancy maternal overweight and obesity [37,38], prenatal maternal gestational diabetes [175], prenatal maternal diet quality [176], and higher omega-6:omega-3 PUFA ratio in cord plasma at birth [177] have been associated with ADHD. Furthermore, as discussed above, dietary interventions have been shown to contribute to improvements in ADHD symptoms [155,173]. Collectively, these findings indicate that nutritional deficiencies and related metabolic factors such as inflammation stemming from poor diet and compromised maternal health may be contributing factors in the development of ADHD. Further supporting the role of diet in the aetiology of ADHD, poor dietary patterns have the potential to disrupt the gut microbiome, triggering chronic inflammation, oxidative stress, and neuroinflammation, all of which have been implicated in the pathophysiology of ADHD [47].

The mechanisms connecting diet and ADHD are complex, involving not just gut and inflammatory factors, but also changes in neurotransmitters and brain functioning. For instance, both zinc and magnesium are important cofactors in neurotransmitter metabolic pathways, potentially explaining the connection between nutritional deficiencies and ADHD symptoms [178]. Vitamins B9 and B12 are essential to the development, differentiation, and function of the central nervous system [179]. Iron deficiency, and in particular low brain iron levels, may impact dopaminergic neurotransmission [100] and neural development, which may result in smaller regional brain volumes [101]. Smaller left hippocampal volume has also been associated with a predominantly Western dietary pattern [133]. Additionally, excess sugar consumption, a potent stimulus for dopamine release, may also contribute to ADHD [180]. The focus of this section, however, will be exploring the interplay among the gut microbiome, inflammatory pathways, and nutritional factors in relation to ADHD.

The gut microbiome, comprising trillions of microbiota and their collective genetic material, plays an essential role in human development, health, and disease [181], supporting critical biological functions not encoded by the human genome [182]. Microbes break down indigestible food compounds like polysaccharides and resistant starches, increasing nutrient availability [183]. Their metabolites, including sugars, short chain fatty acids (SCFAs) such as acetate, propionate, and butyrate, vitamins, and neurotransmitters, are crucial for energy production, immune function, gastrointestinal health, intestinal barrier integrity, signalling, and pathogen inhibition [181,184]. The gut microbiome forms part of the two-way communication system between the microbiota and the brain called the microbiome gut–brain axis, which integrates complex neural, hormonal, immune, and metabolic channels [185].

Gut dysbiosis, a state of pathologically altered gut microbiota, has been shown to have profound impacts on brain function and both mental and physical health. This occurs through various processes that disrupt normal homeostatic functioning, such as the production of harmful metabolites, compromised intestinal barrier integrity, and increased inflammation [182,185,186]. Dysbiosis can be temporary or persistent, is triggered by factors such as unhealthy diet, stress, infection, and antibiotic use, and is implicated in metabolic and inflammatory disease, cancer, and some psychiatric conditions [49,185,187,188]. Studies have identified differences in gut microbiota between individuals with ADHD and healthy controls, with some variations associated with the severity of ADHD symptoms [189,190,191,192,193,194,195]. Some of these differences reflect compositional signatures of dysbiosis, such as a lower abundance of anti-inflammatory bacteria like *Faecalibacterium prausnitzii* [190,193,196] and an increased Firmicutes to Bacteroidetes (F/B) ratio, which has been associated with inflammation [197,198,199].

Diet is a major determinant of gut microbiota composition, with variations in dietary patterns strongly influencing microbiome diversity and balance [200]. For example, greater adherence to the MedDiet or other diets rich in healthy plant-based foods are associated with lower levels of pathogenic bacteria and higher levels of beneficial bacteria, SCFAs, and microbial genes essential for vital metabolic processes [201,202,203,204,205,206,207]. In contrast, opposing associations have been observed from adherence to less healthy diet patterns (e.g., characterised by high intake of SSBs and processed meats) [202,206]. Conceivably, the differences in the gut microbiome observed in individuals with ADHD may reflect the generally lower-quality diets consumed by this population.

Over- or underconsumption of various food groups and nutrients characteristic of modern highly processed diets and often seen in individuals with ADHD has been linked with changes in gut microbiota composition and increased inflammation. Diets high in saturated fat have been shown to alter bile acid composition, disrupt microbial balance, and trigger a pro-inflammatory immune response in susceptible mice [208]. Moreover, a high-fat diet in mice also altered bacterial function and composition, including bacterial diversity and the proportions of the bacterial families *Ruminococcaceae* (decreased) and *Rikenellaceae* (increased) [209]. Another mouse study found that gut bacteria interacted with a high fat diet to promote intestinal inflammation, which did not occur in germ-free (sterile) mice [210]. Collectively, these preclinical studies support a causal link among dietary fat, dysbiosis, and the resultant inflammatory responses.

A lack of dietary fibre has also been found to impact microbiota composition. In healthy, non-obese adults, those with a high saturated fat intake also had a low fibre intake, with this dietary pattern associated with a greater abundance of bacterial taxa potentially associated with obesity and other inflammatory conditions [211]. A study of overweight pregnant women found that low intake of fibre may promote an overgrowth of bacteria favouring lactose degradation, including *Collinsella,* which has been positively associated with circulating insulin, in contrast to the SCFA-producing bacteria promoted by a high-fibre diet [212]. Other specific deficiencies observed in individuals with ADHD may contribute to a pro-inflammatory state and oxidative stress. For example, zinc deficiency can impair immune function, prolong inflammation, and affect gut development, potentially disrupting gut–brain interactions [213]. Magnesium deficiency may contribute to oxidative stress, disturb essential fatty acid metabolism, and impact the levels of the neurotransmitters serotonin, dopamine, and norepinephrine [103]. Vitamin D deficiency may play a role in neuroinflammation and oxidative stress [214]. Omega-3 PUFAs help modulate gut microbiota composition, regulate SCFA levels, and reduce pro-inflammatory mediators [215]. Omega-3 deficiency can induce neuroinflammation [216], while supplementation has been shown to increase beneficial gut bacteria [217,218].

The consumption of food additives and excess refined sugar, common in Western-style diets and potential contributors to ADHD, may similarly be damaging to the gut microbiota. In studies of mice, dietary sugar was found to displace a beneficial bacterium that protects against diet-induced obesity and metabolic disease [219], and high dietary sugar and fat induced gut dysbiosis, damaged the intestinal tract, and altered neurotransmitter metabolism in the brain and intestine, consequently altering brain function [220]. The impact of various food additives such as artificial sweeteners, sugar alcohols, emulsifiers, food colours, flavour enhancers, thickeners, anticaking agents, and preservatives on the gut microbiota has been found to be mostly negative [221]. Titanium dioxide and silica altered the gut microbiota, reduced intestinal barrier function, and increased lipopolysaccharides, with consequent activation of inflammatory factors [222]. Additionally, some gut microbes have been found to be highly susceptible to damage from preservatives such as sodium nitrite, with some anti-inflammatory species being significantly more susceptible than some pro-inflammatory species [223]. Finally, the consumption of gluten and fermentable oligo-, di-, and monosaccharides and polyols (FODMAPS) may also alter gut microbiota in those with a non-celiac gluten sensitivity [224], demonstrating how some naturally occurring food substances may contribute to clinical outcomes via the gut microbiome in those with sensitivities.

**Figure 1:**
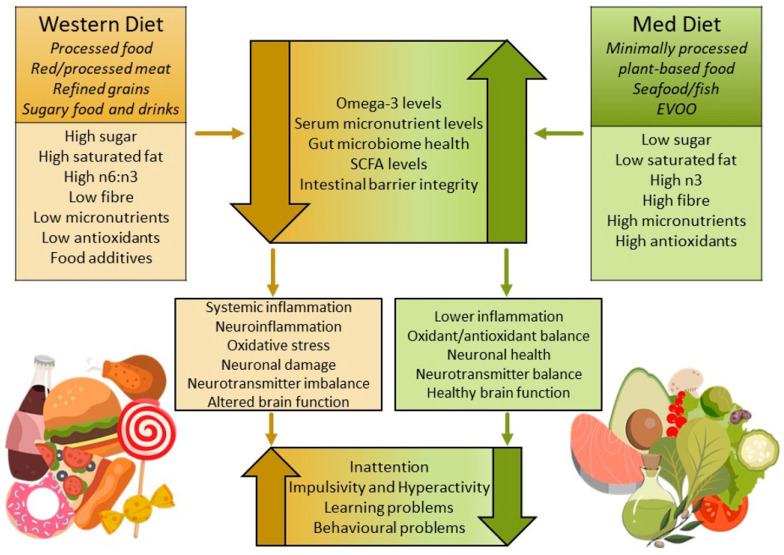
Proposed mechanisms linking diet quality to ADHD pathology and symptom severity. Med: Mediterranean, EVOO: extra-virgin olive oil, n3: omega-3, n6: omega-6, SCFA: short-chain fatty acid.

Research suggests that unhealthy dietary patterns and nutrient deficiencies can significantly alter the gut microbiota, triggering inflammation and other metabolic disturbances. These effects may promote pro-inflammatory taxa and inhibit anti-inflammatory taxa, resulting in reduced SCFA production and increased intestinal barrier permeability. In ADHD, chronic inflammation and persistently increased levels of pro-inflammatory cytokines may weaken the blood–brain barrier, leading to neuroinflammation, or the inflammation of neural tissue [225]. Neuroinflammation triggers the release of harmful oxidative products from brain immune cells [226], resulting in chronic oxidative stress [227], further leading to protein and lipid oxidisation, cell membrane damage, neuronal deterioration, and impaired brain function [65,227,228], which may lead to symptoms of ADHD [47]. Figure 1 illustrates a conceptual outline of the proposed pathways linking dietary patterns, alterations in the gut microbiome, inflammation, and the development of ADHD symptoms.

The potential causative role of diet and gut dysbiosis in ADHD may begin as early as during prenatal development, with maternal diet and nutritional status shown to influence the composition and health of the infant microbiome [229,230,231]. Interestingly, a study in lactating mice has shown that a low-fibre diet can induce gut dysbiosis and inflammation in offspring [232]. Such perturbations in the infant gut microbiome (driven by the maternal diet during pregnancy or lactation) and subsequent inflammation may then go on to adversely influence neurodevelopment. A large cohort study of mother–child pairs investigated associations between measures of ADHD in children and maternal diet quality based on dietary principles consistent with a MedDiet during pregnancy [176]. Maternal diet quality was inversely associated with child ADHD diagnosis and ADHD symptom severity at 8 years of age. Interestingly, child diet quality at 3 years had no impact on measures of ADHD [176]. Maternal adherence to the MedDiet in early pregnancy has also been linked to positive neurodevelopmental outcomes in children. These include reduced incidence of depressive, anxiety, maladaptive, and atypical behaviours, as well as increased likelihood of social relatedness behaviours in the second year of life [233]. Furthermore, adherence to this dietary pattern has been linked to a lower risk of attention-deficit/hyperactivity, oppositional defiant, and depressive symptoms at age 4 years [234]. Prenatal exposure to gestational diabetes mellitus, which may expose the foetus to chronic inflammation, also increases the risk of ADHD [175], reinforcing the potential effects of maternal inflammation on child neurodevelopment.

In contrast to these findings however, a recent prospective cohort study reported that, while prenatal adherence to a MedDiet was associated with a reduced overall risk of neurodevelopmental disabilities in children, no such association was observed specifically for ADHD [235]. Furthermore, an RCT investigating daily DHA supplementation during pregnancy found no significant benefit regarding cognitive measures and no difference in neurodevelopmental diagnoses in children at either 4 [236] or 7 [237] years of age. A separate prenatal DHA RCT found some improvements in sustained attention at 5 years but no effect on other measures of cognitive behavioural or attentional outcomes [238]. In both RCTs, however, mothers only received supplementation in the second half of pregnancy, which may have influenced outcomes.

### 3.2. MedDiet Modulation of Gut Microbiome and Inflammatory Pathways

The MedDiet is relatively understudied in ADHD, especially compared to elimination or supplementation approaches. Given ADHD’s links to dysbiosis, inflammation, oxidative stress, and nutrient deficiencies, the MedDiet—high in anti-inflammatory and antioxidant-rich nutrients, while avoiding harmful components found in highly processed foods—may offer potential benefits for managing ADHD. This section briefly explores evidence for the benefits of the MedDiet and its key components in relation to gut microbiome and anti-inflammatory effects. Table 2 provides a thematic overview of these benefits identified in the research, along with specific findings discussed in the following sections.

#### 3.2.1. The MedDiet

Adherence to a MedDiet has been associated with beneficial changes in gut microbiota composition, including lower *Escherichia coli* counts, a higher *Bifidobacteria:E. coli* ratio, and increased *Candida albicans* and total bacteria and SCFA levels [203]. Intervention studies have further shown that a MedDiet can improve gut microbiota profiles [239] and reduce inflammation and oxidative stress [76,240,241]. For example, a 6-week MedDiet intervention in Crohn’s disease patients resulted in a trend towards microbiota normalisation, significant changes in gene expression, and reduced inflammatory biomarkers [242].

**Table 2:** Thematic overview of physiological benefits of the Mediterranean diet and related dietary features.

Dietary Feature	Physiological Effect	Key References
High MedDiet adherence	**Anti-Inflammatory and Antioxidant Effects**	
	Reduced inflammation and oxidative stress	[76,240,241,242]
	**Gut Microbiota Composition and SCFA Production**	
	Improved gut microbiota composition	[77,203,205,239,242,243,244]
	Reduced *Escherichia coli* and *Ruminococcus gnavus* [203,244] abundance	[203]
	Increased *Bifidobacteria*: *E. coli* ratio, *Candida albicans,* and total bacteria	
	Increased *Faecalibacterium prausnitzii* [205,244], *Eubacterium eligens*, and *Bacteroides cellulosilyticus* abundance	[205]
	Increased *Prevotella* abundance	[77,243]
	Increased levels of total SCFAs	[203]
	**Enhanced Microbial Fibre Metabolism**	
	Enhanced functions for fibre degradation	[205]
	Enhanced genes for microbial fibre degradation	[244]
Fibre/prebiotics	**Gut Barrier Integrity and Inflammation Regulation**	
	Reduced dysbiosis-induced gut permeability and chronic inflammation	[245]
	**Gut Microbiota Composition and SCFA Production**	
	Improved gut microbiota composition and increased levels of SCFAs	[246,247,248]
	Increased *Bacteroidetes* and *Lactobacillus* abundance Reduced *Firmicutes* and *Fusobacterium* abundance	[245]
Polyphenols	**Anti-Inflammatory and Antioxidant Effects**	
	Reduced oxidative stress and DNA damage Scavenging of harmful reactive species Inhibition of enzymes in inflammatory pathways Reduced plasma inflammatory markers	[249,250,251,252]
	**Gut Microbiota Composition**	
	Improved gut microbiota composition	[251,253,254,255]
	Increased *Lactobacilli* and *Bifidobacterial* abundance Reduced *Clostridia* abundance	[251]
Plant-based foods	**Gut Microbiota Composition**	
	Positive association with *Eubacterium eligens* Negative association with *Flavonifractor* and *Ruminococcus torques*	[202]
	Decreased F/B ratio and increased bacterial diversity	[256]
EVOO	**Inflammation, Oxidative Stress, and Apoptosis Regulation**	
	Reduced markers of inflammation and oxidative stress Increased anti-inflammatory factors	[257]
	Inhibition of inflammation, oxidative stress, and apoptosis	[258]
	**Gut Microbiota Composition**	
	Beneficial changes in gut microbiota composition	[259]
	Increased beneficial lactic acid-producing gut bacteria	[257]
Omega-3 PUFAs	**Inflammation and Oxidative Stress Regulation**	
	Reduced plasma inflammatory mediators and oxidative stress	[260]
	Improved inflammatory status	[261,262]
	Inhibition of pro-inflammatory cytokines	[263]
	Reduced metabolic endotoxemia and inflammation	[264]
	**Gut Microbiota Composition and SCFA Production**	
	Increased abundance of SCFA-producing bacterial genera	[265,266]
	Reduced fatty-liver associated genus Increased bacterial fermentation products	[266]
	Increased total and caecal SCFAs	[263,267]
	Increased bacterial alpha diversity	[267]
	Altered gut microbiota composition, with increased beneficial species and reduced pro-inflammatory strains	[264,268]
	**Gut Barrier Integrity and Immune Regulation**	
	Increased tight junction protein expression	[263]
	Improved intestinal barrier integrity	[264,267,268]
	Improved immune homeostasis and metabolic profile	[268]

As a rich source of dietary fibre, the MedDiet encourages proliferation of fibre-fermenting gut microbiota, and thus the production of abundant health-promoting SCFAs [204]. Greater adherence to the MedDiet has been linked to increased abundance of the SCFA-producing species *Faecalibacterium prausnitzii*, *Eubacterium eligens*, and *Bacteroides cellulosilyticus*, alongside enhanced fibre-degrading functions [205]. In a 12-month intervention in firefighters, higher adherence to the MedDiet was associated with multiple bacterial genera involved in SCFA production, particularly *Prevotella*, which is characteristic of plant-rich diets [77] and was similarly enriched following another MedDiet trial [243]. A separate intervention reported increased *F. prausnitzii* abundance, upregulation of microbial fibre-degradation genes, and reductions in potentially pro-inflammatory *Ruminococcus gnavus* and blood cholesterol levels [244]. Additionally, enhanced microbiome gene richness was linked to lower systemic inflammation [244].

#### 3.2.2. Fibre, Polyphenols, and Other Antioxidants

Polyphenols, abundant in fruits, vegetables, whole grains, spices, and legumes, possess powerful antioxidant and anti-inflammatory properties [249]. Approximately 90–95% of dietary polyphenols are metabolised by gut microbiota into bioavailable metabolites [269], which have demonstrated anti-inflammatory activity such as reduced oxidative DNA damage [249]. Flavonoids, the most abundant dietary polyphenols, exert these effects by scavenging harmful reactive species and inhibiting pro-inflammatory enzymes, among other mechanisms [250].

Similar to MedDiet interventions, clinical studies focusing on specific dietary components rich in fibre and polyphenols have demonstrated gut and anti-inflammatory benefits. For instance, whole grain compared to refined grain consumption has been shown to modestly improve gut microbiota composition and enhance SCFAs and immune response indicators [246]. Fibre-rich supplements have produced favourable shifts in gut microbiota among low-fibre consumers [247,248]. A cocoa-derived flavanol intervention significantly increased *Lactobacilli* and *Bifidobacterial* populations, significantly decreased *Clostridia*, and reduced plasma inflammatory markers [251]. In children with ADHD, a bioflavonoid extract normalised urinary catecholamine concentrations—significantly reducing dopamine and non-significantly reducing adrenalin and noradrenalin—whilst also reducing oxidative stress and hyperactivity symptoms compared to placebo [252,270]. In rats fed a high-fat diet, an apple polysaccharide extract modulated dysbiotic gut bacteria by increasing *Bacteroidetes* and *Lactobacillus* while lowering *Firmicutes* and *Fusobacterium* [245]. Dysbiosis-induced gut permeability and chronic inflammation were also reduced [245].

Intervention studies have also revealed positive benefits on gut microbiota composition from consumption of walnuts [253], a wild blueberry drink [254], and broccoli [255]. Healthy plant-based food groups (garnish vegetables, salad, green beans, chard, spinach, fruits, and nuts) have been positively correlated with beneficial bacterium *Eubacterium eligens* and negatively correlated with *Flavonifractor*, a genus linked to poor health, and *Ruminococcus torques* [202], a mucolytic species identified in Crohn’s disease [271] and ASD [272]. In mice, cruciferous vegetables mitigated high-fat-diet-induced inflammation and dysbiosis by decreasing the F/B ratio and increasing bacterial diversity [256].

#### 3.2.3. Extra-Virgin Olive Oil

EVOO, the primary fat in the MedDiet, is a functional food rich in healthy components like monounsaturated fatty acids and phenolic compounds, in contrast to refined olive oils, which are heavily depleted of phenols [273]. EVOO has been shown to play a key role in the anti-inflammatory properties of the MedDiet. After a 3-month MedDiet intervention enriched with EVOO, both overweight/obese participants and healthy weight controls showed significantly reduced markers of inflammation and oxidative stress and increased beneficial lactic acid gut bacteria, whilst anti-inflammatory factors increased in cases [257]. In vivo, phenol-rich EVOO inhibited inflammation, oxidative stress, and apoptosis, protecting against intestinal damage caused by necrotising enterocolitis [258].

Not only the healthy fat content, but also the phenolic compounds in EVOO, are vital to modulating gut microbiota and enhancing inflammatory and oxidative balance. For example, mice fed a high-butter diet developed an obesity-related microbiota profile, while a high-EVOO diet induced the opposite effect, and refined olive oil had an intermediate impact [259], whilst microbiota differences correlated with host physiology [273]. An ex vivo study further showed that an EVOO extract rich in the phenolic compounds secoiridoids exerted anti-inflammatory effects on cells from obese children, while a low-polyphenol olive oil extract did not differ from the control [274].

#### 3.2.4. Omega-3 PUFAs

Omega-3 PUFAs, sourced from key MedDiet components including fish, nuts, and seeds, are well known for their anti-inflammatory properties. In an 8-week trial, omega-3 supplementation in children with ADHD decreased plasma inflammatory mediators, oxidative stress, and hyperactivity scores compared to placebo [260]. Similar improvements in inflammatory status have been observed in patients with ischemic heart failure [261] and metabolic syndrome [262]. Omega-3s also positively influence the gut microbiome. A cross-over trial demonstrated increased abundance in several SCFA-producing bacterial genera following two EPA/DHA supplement formulations [265], while another trial reported elevated beneficial genera and bacterial fermentation products and reduced levels of a fatty-liver-associated genus after omega-3 supplementation [266].

Preclinical studies shed light on mechanisms through which omega-3 intake modulates the gut microbiome in exerting anti-inflammatory effects. In a colitis mouse model, algal-oil-derived DHA altered gut microbiota composition, increased caecal SCFAs and tight junction protein expression, and inhibited pro-inflammatory cytokines [263]. In an in vitro human gut microbial ecosystem, EPA and DHA increased bacterial alpha diversity, altered bacterial abundances, and enhanced total SCFA levels and intestinal barrier integrity [267]. In mice, an anti-inflammatory diet enriched with inulin and omega-3 PUFAs improved intestinal barrier integrity, immune homeostasis, metabolic profile, and gut microbiota composition by increasing beneficial species and reducing pro-inflammatory strains [268]. The benefits of omega-3 PUFAs extend beyond direct gut interactions; mice genetically modified to maintain high tissue levels of omega-3 have demonstrated altered gut microbiota composition, reduced intestinal permeability, and lower levels of metabolic endotoxemia and inflammation—effects also replicated in non-modified mice supplemented with omega-3 PUFAs [264]. These findings reinforce the role of omega-3s in supporting gut barrier function and reducing inflammatory responses.

## 4. Limitations and Future Directions

Multiple observational studies have consistently reported that both maternal and individual dietary patterns associated with ADHD tend to be of lower nutritional quality. Combined with evidence of nutrient deficiencies and symptomatic improvement from dietary interventions, these findings suggests that diet may play a contributing factor in ADHD development. However, the reliability of this conclusion is tempered by inconsistencies across studies and the complex relationship between ADHD symptoms and food consumption. For instance, symptoms of ADHD have been found to positively correlate with binge/disinhibited and restrictive eating and decreased awareness of internal signals of hunger and satiety, which also positively correlate with disordered eating [275]. Greater emotional symptoms and hyperactivity are also associated with greater emotional overeating in children [276]. This suggests that excessive and other unhealthy forms of eating in ADHD could result from impulsivity, lack of body awareness, and emotional dysregulation, which are important features of ADHD.

Those with ADHD may also experience distraction when eating, forget they have eaten and therefore go on to consume additional highly palatable food [275], or be particularly selective eaters, leading to less nutritious, restricted diets [277]. The dopaminergic system, affected in ADHD, is involved in the processing of food as a reward [278] and therefore the degree of palatability of a food and its role in reinforcement of particular eating behaviour may be enhanced in ADHD. This may be especially relevant with sugar, since its ability to stimulate dopamine release [180] may be inadvertently utilised to reverse dopamine deficiency. The high heritability of ADHD and the resulting increased likelihood that parents of individuals with ADHD also have deficits in self-control and impulsivity may contribute to an environment with greater availability of unhealthy convenience or snack foods, as well as a family pattern of disordered eating [137]. This may also partially explain links between the observed poor maternal nutritional status and greater chance of ADHD in offspring. Finally, a common side effect of stimulant medication is appetite suppression [279], which could play a role in inhibiting the intake of complete meals and reliance on nutrient-devoid smaller snacks.

To fully understand the role of diet in ADHD and its potential causative impact, further robust dietary intervention research is needed. Such interventions could be implemented prenatally, during lactation, and in early life to determine how nutrition may influence the development of ADHD and the most critical periods in this process. Alongside clinical and dietary assessments, measures of the gut microbiome and inflammation during these early developmental periods would help clarify the mechanisms linking nutritional factors and clinical outcomes. Further, given the complexity and heterogeneity of ADHD, precision nutrition (or personalised nutrition) may indeed be an attractive lifestyle approach for the management of ADHD symptoms. Although there is no universally accepted definition for precision/personalised nutrition, these terms are often used interchangeably [280], although some authors have attempted to make distinctions between the two terms [281]. Nevertheless, irrespective of its operationalisation, both precision and personalised nutrition aim to use personal information about an individual (or cohort of people) to deliver nutritional advice that, theoretically, is more suitable for them compared with generic advice, which may aid in better dietary compliance, symptom management, and attenuation of disease progression. However, such literature is currently lacking in ADHD.

Additional MedDiet interventional research with adequate statistical power is warranted, in particular in ADHD, due to the demonstrated potential of this dietary pattern to address deficiencies, reduce harmful food substances, and improve the gut microbiome and inflammatory statuses. This could also be carried out in the adult ADHD population, which to date has been largely ignored in dietary research despite symptoms persisting into adulthood for a large number of affected individuals. Although some individuals may improve on a restricted diet due to sensitivities, these diets may not meet the individual’s nutritional requirements, so long-term adherence may require caution to avoid further deficiencies [282]. For some, a modified MedDiet approach may be feasible, such as a low-gluten or low-salicylate option. Dietary research could also compare the efficacy of a whole-diet approach to individual supplements or dietary components, such as omega-3 PUFAs or EVOO, to ascertain the degree of dietary modification necessary for physiological and clinical improvements. This may be particularly important among youth with ADHD, given the complex relationship around food intake in some members of this population, which may reduce likelihood of adherence to healthy eating and make supplementation more practical. The feasibility and acceptability of sustaining dietary changes in individuals with ADHD is another important question that needs to be addressed, including the extent to which a traditional MedDiet can be realistically accepted, and how much adaptation may be required for consumers from non-Mediterranean countries.

As noted in the section on supplementation in ADHD, future dietary research should establish optimal dosages, as inconsistencies in findings may reflect varying dosages, and also assess pre- and post-intervention nutrient serum levels. This is important in order to determine if specific deficiencies are being addressed, whether an individual with ADHD in fact needs to be deficient in a nutrient to benefit from dietary change and/or supplementation, and if this varies according to the specific nutrient. Research has shown that supplementation can increase nutrient levels in those who are deficient [107,115,116] and that benefits may only achieved or are greatest in those with baseline deficiency [93,104], although those without deficiency can also benefit [105], whilst the majority of interventional research has not assessed serum levels.

## 5. Conclusions

ADHD is a prevalent neurodevelopmental disorder linked to adverse outcomes across the lifespan, arising from complex interactions between genetic and environmental factors. Its pathophysiology involves neurotransmitter imbalances, brain differences, and alterations in the gut microbiota, potentially leading to inflammation and oxidative stress, which may be influenced by poor dietary habits. Although pharmacological treatments are often efficacious, they do not always generate sufficient treatment response, and raise concerns regarding safety, tolerability, and long-term use. As such, alternative approaches, including nutritional psychiatry, are gaining attention. ADHD is linked to particular nutrient deficiencies, including omega-3 PUFAs, zinc, magnesium, iron, vitamin D, and B vitamins. Clinical trials provide support for the role of targeted supplementation in symptom management; however, heterogenous study designs, particularly in dosage and duration, complicate interpretation of findings. Moreover, unhealthy eating patterns such as the Western diet and its components like saturated fats, sugar, and additives have been associated with ADHD diagnosis and severity, while healthy patterns like the MedDiet may offer protective benefits. Whole-diet interventions, primarily involving elimination diets and, to a lesser extent, healthy diets like the MedDiet, have been trialled in ADHD. Whereas elimination diets can benefit individuals with food sensitivities, they may be too restrictive for long-term use, underscoring the need for robust interventional research on the efficacy of healthy dietary patterns in ADHD. Emerging evidence suggests that diet may influence ADHD through gut dysbiosis, impaired intestinal barrier integrity, and resulting systemic inflammation, and oxidative stress, possibly beginning early in development. However, the bidirectional relationship between ADHD and eating behaviours complicates causal interpretations involving diet and pathogenesis. Therefore, further interventional research is needed to clarify the role of diet and the gut microbiome in ADHD pathophysiology. The MedDiet and its key components, such as fibre, polyphenols, other antioxidants, EVOO, and omega-3 PUFAs, have demonstrated beneficial modulation of the gut microbiome and inflammatory processes. It is therefore well-positioned for investigating both the dietary management of ADHD and its underlying biological mechanisms. MedDiet feasibility, including necessary modifications for acceptance by the ADHD population, should also be explored.

## Data Availability

No new data were created or analysed in this study. Data sharing is not applicable to this article.

## Abbreviations

The following abbreviations are used in this manuscript:

ADHD	Attention-deficit/hyperactivity disorder
ASD	Autism spectrum disorder
US	United States
MedDiet	Mediterranean diet
PUFA	Poly-unsaturated fatty acid
EPA	Eicosapentaenoic acid
DHA	Docosahexaenoic acid
RCT	Randomised controlled trial
ARA	Arachidonic acid
SSB	Sugar-sweetened beverage
AFC	Artificial food colouring
CVD	Cardiovascular disease
EVOO	Extra-virgin olive oil
DASH	Dietary Approaches to Stop Hypertension
SCFA	Short chain fatty acid
F/B	Firmicutes to Bacteroidetes ratio
FODMAPS	Fermentable oligo-, di-, and monosaccharides and polyols
